# Poland-Möbius syndrome: a case report implicating a novel mutation of the *PLXND1* gene and literature review

**DOI:** 10.1186/s12887-022-03803-3

**Published:** 2022-12-30

**Authors:** Graeme E. Glass, Shiyas Mohammedali, Bran Sivakumar, Mitchell A. Stotland, Faisal Abdulkader, Debra O. Prosser, Donald R. Love

**Affiliations:** 1 Department of Surgery, Sidra Medicine, Doha, Qatar; 2 Weill Cornell Medical College, Doha, USA; 3 Weill-Cornell Medical College, Ar-Rayyan, Qatar; 4 Qatar Foundation, Education City North Campus, Room C1-120, 1st Floor OPC, Al Luqta Street, PO BOX 26999, Doha, Qatar; 5 Department of Plastic Surgery, Great Ormond Street Hospital for Children NHS Foundation Trust, London, UK; 6 Department of Pathology, Sidra Medicine, Doha, Qatar

**Keywords:** Möbius, Moebius, Poland syndrome, Symbrachydactyly, Pectoralis hypoplasia, Case report

## Abstract

**Background:**

Möbius (Moebius) and Poland’s syndromes are two rare congenital syndromes characterized by non-progressive bilateral (and often asymmetric) dysfunction of the 6^th^ and 7^th^ cranial nerves and hypoplasia of the pectoral muscles associated with chest wall and upper limb anomalies respectively. Manifest simultaneously as Poland-Möbius (Poland-Moebius) syndrome, debate continues as to whether this is a distinct nosological entity or represents phenotypic variation as part of a spectrum of disorders of rhomboencephalic development. Etiological hypotheses implicate both genetic and environmental factors. The *PLXND1* gene codes for a protein expressed in the fetal central nervous system and vascular endothelium and is thus involved in embryonic neurogenesis and vasculogenesis. It is located at chromosome region 3q21-q22, a locus of interest for Möbius syndrome.

**Case presentation:**

We present the first report of a patient with Poland-Möbius syndrome and a mutation in the *PLXND1* gene. A child with Poland-Möbius syndrome and a maternally inherited missense variant (NM_015103.2:ex14:c.2890G > Ap.V964M) in the PLXND1 gene is described. In order to contextualize these findings, the literature was examined to identify other confirmed cases of Poland-Möbius syndrome for which genetic data were available. Fourteen additional cases of Poland-Möbius syndrome with genetic studies are described in the literature. None implicated the PLXND1 gene which has previously been implicated in isolated Möbius syndrome.

**Conclusions:**

This report provides further evidence in support of a role for *PLXND1* mutations in Möbius syndrome and reasserts the nosological link between Möbius and Poland’s syndromes.

**Level of evidence:**

Level V, Descriptive Study.

**Supplementary Information:**

The online version contains supplementary material available at 10.1186/s12887-022-03803-3.

## Background

Möbius (phonetically, Moebius) syndrome is a rare congenital disorder present in an estimated 1 per 50,000 live births and characterized by bilateral (and often asymmetric) facial paralysis with a concomitant bilateral deficit in ocular abduction. Clinically, this manifests as mask-like facies and a bilateral esotropia (convergent strabismus). Difficulties with emotional expression and social adjustment are observed in up to 40% of cases, and the debate continues regarding the extent to which this is a consequence of difficulties with non-verbal expressivity [1]. Additional manifestations may include subtle or obvious deficits in cranial nerves (especially III, IX, X and XII) [2], and cardiovascular anomalies including septal defects, vessel transposition and dextrocardia [3]. Feeding and respiratory problems also appear to be features in some cases and the reason is probably multifactorial neuromuscular dysfunction involving the tongue, face and swallow reflex [4]. Prior reports of intellectual dysfunction in up to 50% of cases is probably exaggerated, not least because of drooling and lack of expressive congruence, with some reports suggesting normal baseline intellect [5], despite problems with social interaction [6]. There is no gender preponderance. The condition has recently been re-defined as essentially a problem of rhomboencephalic development, with motor nuclei and axons predominantly affected [7]. The lack of clearly defined diagnostic criteria continues to present challenges when making large scale predictions about etiology and pathogenesis [8]. Genetic associations with Möbius syndrome (MBS) have been difficult to unravel with several loci implicated including 1p22, 3q21-22, 10q21.3–22.1 and 13q12.2–13 [9]. These locations were termed MBS1-3 and each has a number of candidate genes reviewed by Kadakia and colleagues [10].

Poland’s syndrome is a rare congenital disorder present in around 1 in 30,000 live births and characterized by upper limb and thoracic mal-development [11]. Upper limb anomalies include arm, forearm and hand hypoplasia and variable digit anomalies including hypoplasia, syndactyly, symbrachydactyly, radial or ulnar ray anomalies [12] while the chest wall anomalies exhibit a spectrum of deformity, including absence of the sternal head of pectoralis major at least although in addition hypo or aplasia of the pectoral muscles, serratus anterior and the external obliques with winging of the scapula and thoracic scoliosis have been described. Skeletal chest wall deformity, including pectus excavatum or pectus carinatum is often present as is hypo or aplasia of breast tissue including the nipple areolar complex. Males predominate at a ratio of around 3 to 1 and most, but not all reports suggest that it favors the right side in males [13]. While usually unilateral, bilateral cases have been reported [14]. While familial cases have been described [15–17], there is no clear genetic association.

As upper and lower limb anomalies are a recognized feature of Möbius syndrome in over a third of cases [18], Herrmann and colleagues recognized the nosologic obfuscation involved in describing Möbius, Hanhart and Poland syndrome variants and first proposed the term Poland-Möbius (Poland-Moebius, hereafter referred to as PMS) as a distinct nosological entity [19]. The first description of what would become known as PMS is attributed to Jorgenson, in 1971 [20]. The prevalence of idiopathic or familial Poland-Möbius syndrome (PMS) is roughly 1 in 500,000 live births [21]. Maternal cocaine use [22], misoprostol [23] and *in-vitro* fertilization (IVF) [24] are attributed causes in isolated cases. PMS has been described obliquely in many sources [25–27] and may be present in up to 20% of cases where Möbius syndrome has been diagnosed [28, 29]. A further proportion of cases reside within a nosologic grey area, exhibiting cranial nerve palsies, hemifacial microsomia, facial dysmorphism, micrognathia, auricular deformities, cervical spine and upper limb anomalies and adding credence to the view that Poland and Möbius syndromes are manifestations of a broad canvas of embryologically-related anomalies characterized by chest, spine, upper limb and facial mal-development that includes Hanhart, Sprengel, Klippel-Feil, Pierre-Robin, Goldenhar and Carey-Fineman-Ziter syndromes [7, 30–35].

Establishing an inheritance pattern has been complicated by the fact that most cases appear to occur sporadically, adding weight to the embryonic vascular insult hypothesis. The theory postulates that an embryonic subclavian artery insult at around the 6^th^ week of gestation may account for a number of congenital oculo-maxillofacial, cervical and upper limb anomalies the distinct features of which have been codified in a plethora of eponymous syndromes [36].

While no genetic patterns have yet emerged to support PMS as a distinct entity, the *PLXND1* gene at locus 3q21-22 has recently been implicated in a number of cases of isolated Möbius syndrome [37, 38]. Here, for the first time, we report a case of PMS associated with a novel mutation in the *PLXND1* gene (NM_015103.3) and compare this with the mutations observed in the *PLXND1* gene in isolated Möbius syndrome. Further, we evaluate the literature to place this finding in the context of what is already known about the genetic associations of PMS.

## Case presentation

The child was born at term via elective Cesarean section, the second child born of consanguineous (first cousin) parents from Pakistan. The prenatal history was uneventful and there was no maternal history of medical or recreational drug use. He was noted to have a right sided facial palsy at birth and only later were additional concerns raised about the left side in addition. Further, it was noted that he exhibited an anomaly of his left upper limb with symbrachydactyly and an associated chest wall deformity and apparent absence of pectoralis major and an absent nipple areolar complex. He experienced an episode of milk aspiration and was admitted to neonatal intensive care unit where he stayed for 20 days. Echocardiography revealed a patent ductus arteriosus and a moderate atrial septal defect which was treated expectantly. Oral intake remained poorly coordinated and feeding was supported using a nasogastric tube. Flexible nasal largyngoscopy revealed mild to moderate laryngomalacia and a deep inter-arytenoid groove with salivary pooling. On account of generalized hypotonia he underwent magnetic resonance imaging (MRI) of the brain which revealed an incidental finding of a prominent left frontal developmental venous anomaly. A contrast study for persistent gastro-esophageal reflux revealed an anatomically normal alimentary canal. On account of persistent reflux and a further aspiration, he underwent insertion of a gastric tube with fundoplication.

**Fig. 1:**
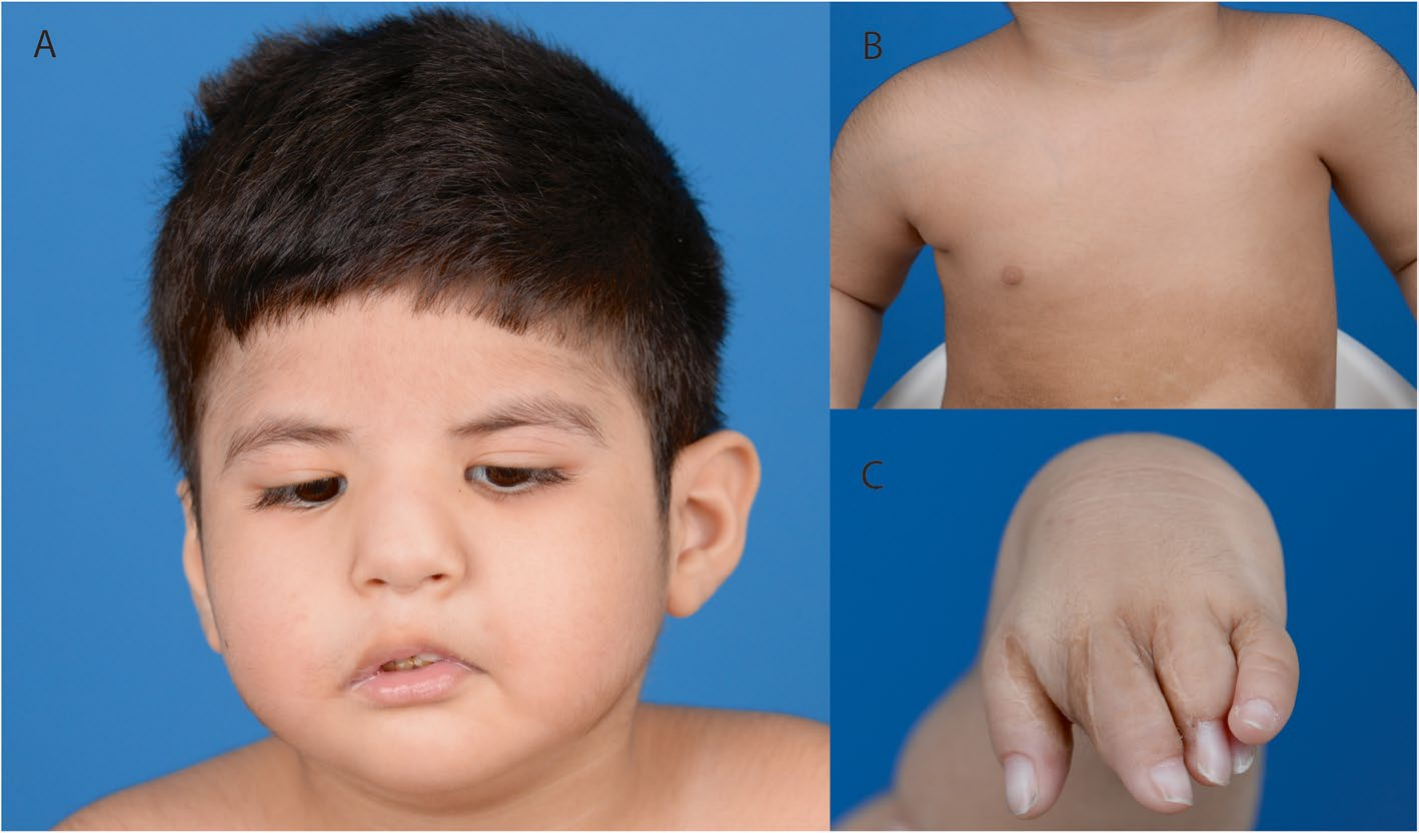
The phenotype of the proband. **A** Facial features, demonstrating a bilateral (asymmetric) facial palsy, convergent esotropia and bilateral blepharoptosis. **B** Left sided chest wall anomalies including absence of pectoralis major and the nipple-areolar complex. **C** Left hand symbrachydactyly following web space release

Within the first 12 months of life he was examined by plastic surgeons specializing in facial palsy (GG) and congenital limb anomalies (BS). Facial examination revealed bilateral, asymmetric facial palsies (worse on the right), bilateral failure of ocular abduction and bilateral blepharoptosis with no skeletal facial dysmorphism. A high arched but intact hard palate was noted. He exhibited good masseteric contractions bilaterally. Examination of the upper limbs and chest revealed a complete absence of pectoralis major and the nipple areolar complex on the left with significant axillary hollowing. Hypoplasia of left upper limb, particularly forearm and hand were noted. Symbrachydactyly was noted, with a reasonable thumb including thenar musculature. Flexion was observed at the metacarpophalangeal and interphalangeal joints. The index, middle and ring fingers were hypoplastic, biphalangeal and syndactylized. Active movement was exhibited at the elbow and shoulder. At 18 months of age he underwent release of the 1^st^ and 4^th^ web space syndactyly. He remains a candidate for bilateral facial reanimation. Based on these findings a diagnosis of Poland-Möbius syndrome was made. The clinical phenotype is shown in Fig. 1.

The genetic workup revealed identified two variants in the *PLXND1* gene; while one was a synonymous, likely benign (LBEN) variant, the other was a missense variant of uncertain significance (VUS) as detailed in Table 1. Familial analysis confirmed the variant to be maternally inherited. Clinical history and examination of the mother revealed that she does not exhibit PMS.

**Table 1:** The *PLXND1* gene variants identified in this patient. RSID: Reference Single Nucleotide Polymorphisms Cluster Identification; PLXND1: Plexin D-1; het: heterozygous; LBEN: Likely benign; VUS: variant of uncertain significance

Gene	Zygosity	Annotations	Type	Position	RSID	gnomAD	Provean	Classification
PLXND1	het	NM_015103.2:ex27: c.4677C > T p.N1559N	synonymous	chr3:129,281,778	rs767882412	0.000112		LBEN
PLXND1	het	NM_015103.2:ex14: c.2890G > A p.V964M	missense	chr3:129,291,732	rs553781761	8.38E-05	-2.14	VUS

### Poland-Möbius (Poland-Moebius) syndrome in the literature

While we identified numerous descriptions of PMS in the literature, and while PMS is described obliquely in many more studies, we found that PMS was both comprehensively described and subjected to genetic evaluation in only 14 cases. The genetic investigation employed was traditional karyotyping or microarray analysis in 13 cases. In one case, a copy number gain in chromosome 3q23 was identified [39]. In another case, a deletion in the *REV3L* gene located at 6q21 was described [40]. In a third case, a chromosomal translocation event involving chromosomes 1 and 11 was reported [41]. In the remaining 11 cases, a normal molecular karyotype was observed and no further genetic analyses were undertaken. These cases are summarized in Table 2. All mutations described in the *PLXND1* gene when implicated in Möbius syndrome or PMS are summarized in Table 3. The systematic review methodology is summarized in the supplementary methods document, in supplementary Fig. 1 and in supplementary tables 1–3.

**Table 2:** A clinical and phenotypic summary of Poland-Möbius syndrome in the literature when comprehensively described and accompanied by genetic studies

Year	Reference	Demographic	Genetic associations	Clinical features
2021	Current paper	Male infant Consanguineous No FHx	Mutation in *PLXND1* gene (NM_015103.2; chr3q22.1) located on chromosome 3q21-q22	*Möbius:* Bilateral facial palsy (R > L) Bilateral abducens palsy Dysphagia, Laryngomalacia
				*Poland (Left):* Hypoplasia of pectoralis major Chest wall deformity Absence of nipple areolar complex Hypoplasia of upper limb Symbrachydactyly of hand
				*Other:* Atrial septal defect Generalized hypotonia
2016	Vaccari et al. [40]	Male infant Consanguinity not stated	Deletion of the *REV3L* gene (NM_002912.5; chr6q21)	*Möbius:* Right facial nerve paralysis; epicanthic folds; micrognathia and cleft palate (Pierre Robin sequence, MIM261800); right abducens nerve paralysis; right auditory nerve paralysis
				*Poland(Right):* Right upper limb hypoplasia Flexion deformity of the left elbow and the left wrist Scoliosis, pectus excavatum
				*Other:* Weight and head circumference < 3rd centile; length at 5th centile Intellectual disability
2013	Flores et al. [39]	Male infant Non-consanguineous No FHx	Microarray only: Gain of 3 Mb on chromosome 3q23	*Möbius:* Right sided facial palsy Bilateral abducens palsy Dysphagia Wide forehead, high arched palate, micrognathia
				*Poland (left):* Absent pectoral muscles Chest wall deformity and defect (lung hernia) Hypoplasia of nipple areolar complex Hypoplasia of upper limb
				*Other:* Dextrocardia Left hydronephrosis Left talipes equinovarus
1993	Donahue et al. [41]	Male infant Non-consanguineous	Karyotype showed a t(1;11)(p22;p13) translocation	*Möbius:* Bilateral facial palsy Bilateral abducens palsy
				*Poland (left):* Absent sternal head of pectoralis major Absent pectoralis minor Syndactyly of ipsilateral hand Hypoplastic nipple
				*Other:* Cleft palate, Dextrocardia, mandibular hypoplasia, and multiple areas of diffuse brain volume loss
2012	Ahmad et al. [42]	Male infant Non-consanguineous No FHx	Microarray only (normal molecular karyotype)	*Möbius:* Left facial palsy Strabismus Ptosis, low set ears, micrognathia
				*Poland (right):* Absent pectoral muscles Hypoplastic upper limb Symbrachydactyly of hand Hypoplastic thumb
				*Other:* Brachycephaly Bilateral talipes equinovarus
2011	Abbas et al. [43]	Male infant Non-consanguineous No FHx	Microarray only (normal molecular karyotype)	*Möbius:* Bilateral facial palsy (L > R) Bilateral abducens palsy High arched palate
				*Poland (right):* Hypoplastic pectoral muscles Hand acheiria
2010	Carolina Cares et al. [44]	Male infant Consanguinity not stated	Normal male karyotype (46XY)	*Möbius:* Left facial palsy Left hemifacial microsomia Left microtia
				*Poland (Left):* Agenesis of left pectoralis, hypoplasia of left radius and hand
				*Other:* Short neck,C4-C5 fusion
2009	Al-Mazrou et al. [24]	Female infant Consanguineous	Normal female karyotype (46XX)	*Möbius:* Bilateral facial palsy
				*Poland (Right):* Hypoplastic right pectoralis major Hypoplastic right upper limb 2nd, 3rd and 4th partial syndactyly and brachydactyly
				*Other:* Left hand digits camptodactyly Bilateral talipes equinovarus Macrocephaly Low set ears
2008	Lopez de Lara et al. [45]	Male adolescent non-consanguineous no FHx	Microarray only (normal molecular karyotype)	*Möbius:* Bilateral facial palsy Bilateral ophthalmoplegia, bilateral ptosis Carp-shaped mouth, high arched palate
				*Poland (left):* Absent pectoralis major and trapezius Cubitus valgus Hypoplastic hand, 5^th^ digit clinodactyly
				*Other:* Hypogonadotrophic hypogonadism Micropenis Psychomotor delay
2005	Puvabandistin et al. [22]	Male infant Consanguinity not stated	Normal male karyotype (46XY)	*Möbius:* Bilateral facial palsy Bilateral abducens nerve palsy bilateral epicanthus, negative canthal axis, micrognathia
				*Poland (Right):* Absent right pectoralis, nipple and areola Right forearm hypoplasia
				*Other:* Atrial septal defect
2004	Dufke et al. [34]	Male child Non-consanguineous	Normal male karyotype (46XY)	*Möbius:* Bilateral facial palsy Bilateral abducens nerve palsy
				*Poland (Right):* Absent right pectoralis muscles ulnar deviation of the right hand
				*Other:* Global developmental delay Pierre Robbin sequence
1999	Larrandaburu et al. [46]	Female adolescent Non-consanguineous Maternal aunt with Poland Syndrome	Microarray only (normal molecular karyotype)	*Möbius:* Bilateral facial palsy Bilateral convergent strabismus
				*Poland (right):* Aplasia of sternal head of pectoralis major, aplasia of pectoralis minor Aplasia of breast Hand symbracydactyly, triphalangeal thumb
				*Other:* Severe psychomotor delay
1997	Matsui et al. [47]	Male child Consanguinity not stated No FHx	Microarray only (normal molecular karyotype)	*Möbius:* Bilateral facial palsy Bilateral abducens palsy Esotropia right eye
				*Poland (Right):* Aplasia of pectoral muscles with associated chest wall defect only
				*Other:* Right bundle branch block ASD with significant left to right shunt
1984	Bosch-Banyeras et al. [48]	Male infant Non-consanguineous No FHx	Microarray only (normal molecular karyotype)	*Möbius:* Bilateral facial palsy Convergent strabismus Dysphagia
				*Poland (left):* Hypoplasia of pectoralis major Chest wall deformity and defect (lung hernia) Absence of nipple areolar complex Hypoplastic upper limb Hand acheiria
				*Other:* Dextrocardia
1981	Parker et al. [49]	Male adolescent Non-consanguineous	Microarray only (normal molecular karyotype)	*Möbius:* Bilateral facial palsy Bilateral abducens palsy Micrognathia, bilateral ear anomalies, tongue atrophy & weakness
				*Poland (left):* Absent pectoralis major and minor Hypoplastic left arm Symbrachydactyly of hand, rudimentary thumb
				*Other:* Left talipes equinovarus, hypoplastic leg and foot

*FHx* Family history, *R* Right, *L* Left; *PLXND1* Plexin D-1, *ASD* Atrial Septal Defect

**Table 3:** A summary of the *PLXND1* gene variants described in the literature in patients with Möbius syndrome

Patient	PLXND1 mutation
1	c.5685C > A; p.Asn1895Lys
2	c.4454_4455GC > CA; p.Arg1485Pro
3	c.3018C > T; p.Leu1006Leu
4 (our patient)	c.2890G > A; p.Val964Met

## Discussion

This paper describes a novel mutation in the *PLXND1* gene at locus 3q21-22 in a patient with Poland-Möbius syndrome. In doing so it provides additional evidence to support the view that the chromosomal locus 3q21-23 is an area of interest as we seek a genetic association for Möbius, Poland-Möbius, and associated eponymous syndromes. Furthermore, it provides circumstantial evidence to support the view that both Poland and Möbius syndromes are different manifestations of essentially the same disorder of rhomboenecephalic development.

In 1996 Kremer and colleagues identified the chromosomal locus 3q21-22 (MBS 2) as one of 3 candidate loci for Möbius syndrome, based on an extensive family study of familial Möbius syndrome exhibiting an autosomal dominant inheritance pattern [50]. The others are 13q12.2–13 (MBS 1) [51, 52] and 10q21.3–23.1 (MBS 3) [53] respectively, suggesting genetic heterogeneity. In 2002, van der Zwaag and colleagues proposed *PLXND1*, present within the candidate locus 3q21-22, as a candidate single gene cause for Möbius syndrome [9], based on studies that observed *PLXND1* gene expression in embryonic central nervous system (including cranial ganglia) and vascular endothelial cells. The same group examined candidate genes from the MBS 2 and 3 loci [54], including *PLXND1* [55] without establishing definitive evidence of any causative mutations. *PLXND1* was revisited in 2015 with the publication of a multicenter study that included additional genetic study data from the original PLXND*1* study cohort. On this occasion, the larger study cohort of MBS yielded 3 de novo* PLXND1* gene mutations and 3 additional de novo mutations in the gene *REV3L* one of whom exhibited incidental features of Poland’s syndrome [38]. The authors highlighted the diagnostic yield offered by whole exome and whole genome sequencing of parent-patient trios. In our study, reflex analysis of the parents confirmed the heterozygous missense variant in the *PLXND1* gene variant in our patient was inherited from the mother (who was also a carrier), hence not de novo. We did not undertake segregation analysis of the heterozygous likely benign variant in the *PLXND1* gene. Taken in isolation, this suggests that while the missense variant in the *PLXND1* gene is novel, its causal relationship to Mobius syndrome may not be strong. It is unclear, however, if reduced penetrance may be playing a role and so the detected variant may still be clinically significant. The variant *p.Val964Met* lies within the amino-terminal IPT (Immunoglobin-like fold shared by Plexins and Transcription factors) domain. Tomas-Roca et al. suggest that there is evidence that these domains are functionally important, and that a missense mutation in one of the domains in *PLXND1* may lead to an inactive receptor. It is also unclear if parent-of-origin effects may be playing a role as the proband’s mother is unaffected. A larger family study could address this possibility. It should nonetheless be of considerable interest to both clinical researchers and geneticists that our paper is the first to describe both Poland and Möbius syndrome in a patient with a mutation in the *PLXND1* gene.

PMS has also been described in association with dextrocardia in 3 cases [39, 48, 56] and in one such case, microarray revealed a gain in location 3q23, adjacent to the 3q21-22 locus of interest. While this observation does not provide proof of an association, it reaffirms the view that this region is a chromosomal loci of interest in the complex process of unravelling the genetics of MS and PMS.

Only one familial case has been described; a child with PMS born to a mother with Poland’s syndrome. Unfortunately, no genetic work up was conducted [57]. Again, however, this is suggestive of an inextricable link between these syndromes and requires us also to look at the evidence for genetic associations with Poland syndrome. Of the candidate genes thus far implicated in Poland syndrome, only *REV3L* (chromosomal locus 6q21-22.1) appears to be of interest here (see Table 2) [40].

The main drawback of this paper is that, as the carrier mother did not express the phenotype, the link between *PLXND1* and MS or PMS remains unproven and our observation simply adds further weight to the body of circumstantial evidence linking MS and PMS to the 3q21-22 locus and, perhaps, the *PLXND1* gene. However, it is from the cumulative body of observational data that a hypothesis will emerge and thus the next step is further preclinical analyzes of the 3q21-22 locus which are ongoing.

## Conclusion

This paper describes a case of a child with Poland-Möbius syndrome in the context of a maternally inherited *PLXND1* gene mutation, a gene that is considered a candidate gene for Möbius syndrome but has hitherto not been described in the context of either Poland syndrome or Poland-Möbius syndrome, despite multiple case reports suggesting a nosologic link between the two syndromes. This report provides further circumstantial evidence of a causal link but suggests either that penetrance varies widely between cases or that, as yet further unidentified causal variables make crucial contributions to phenotypic expression in genetically predisposed individuals.

## Supplementary Information

**Additional file 1:** **Supplemental figure 1.** A summary of the literature search and study selection.

**Additional file 2:** **Supplemental table 1.**  CARE checklist.

**Additional file 3:** **Supplemental table 2.** Search  strategy.

**Additional file 4:** **Supplemental table 3.** PRISMA  checklist.

## Data Availability

All relevant supporting information can be found in supplemental Fig. 1 and supplemental Tables 1–3. The datasets generated and/or analyzed during the current study are available in the ClinVar repository and may be found at the following address: https://www.ncbi.nlm.nih.gov/clinvar/variation/1800855/?new_evidence=true. The variant ID is: 1,800,855.
