# Supplementary figures and images for: Poland-Möbius syndrome: a case report implicating a novel mutation of the PLXND1 gene and literature review
Source: BMC Pediatr. 2022 Dec 30;22:745. doi: 10.1186/s12887-022-03803-3 (PMC9801559; doi:10.1186/s12887-022-03803-3)

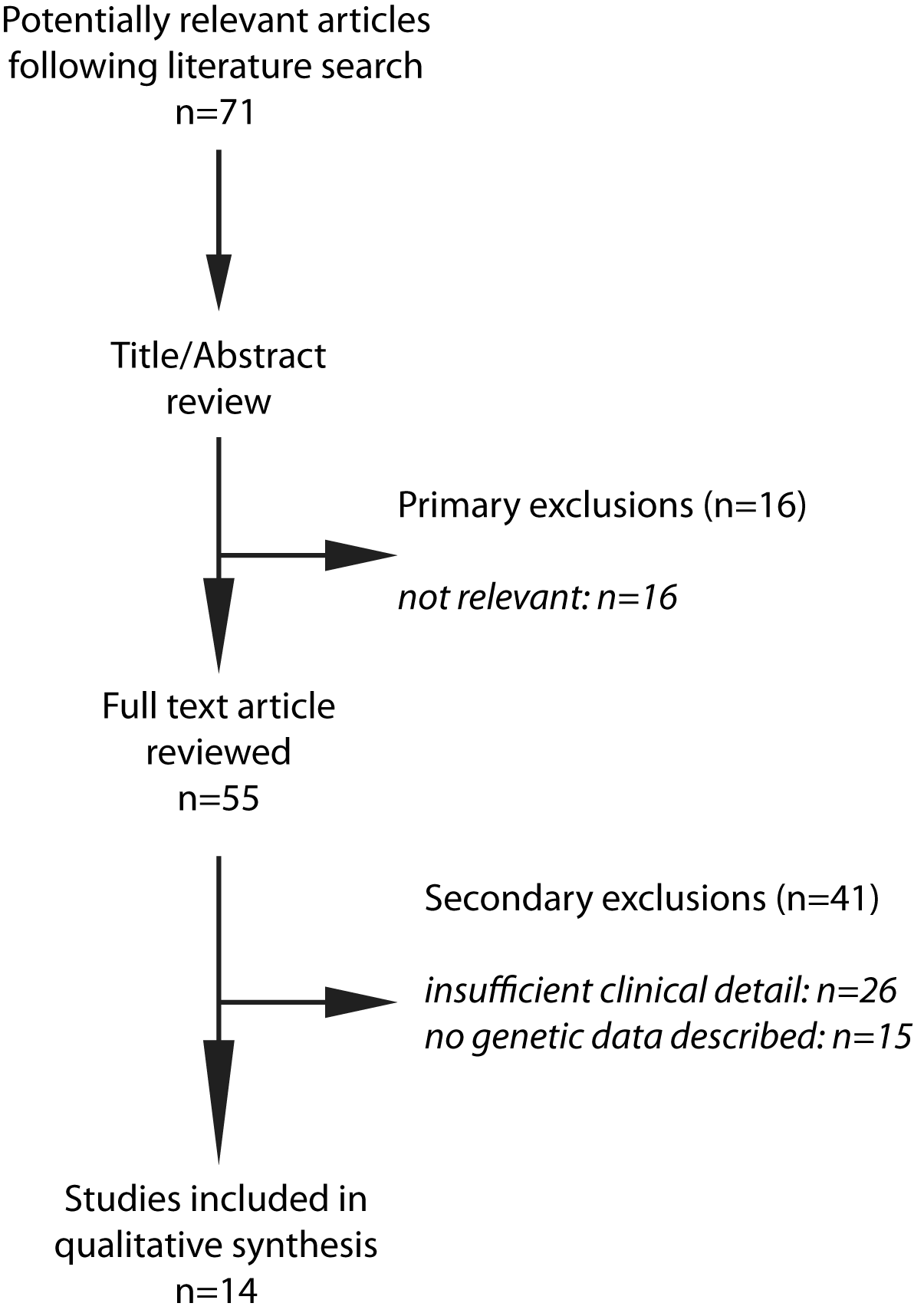

Supplement: Supplementary file 1 — Additional file 1: Supplemental figure 1. A summary of the literature search and study selection. [file 12887_2022_3803_MOESM1_ESM.tif]
