# Supplementary material for: Poland-Möbius syndrome: a case report implicating a novel mutation of the PLXND1 gene and literature review
Source: BMC Pediatr. 2022 Dec 30;22:745. doi: 10.1186/s12887-022-03803-3 (PMC9801559; doi:10.1186/s12887-022-03803-3)
Supplement: Supplementary file 3 — Additional file 3: Supplemental table 2. Search strategy. [file 12887_2022_3803_MOESM3_ESM.pdf]

Pubmed Search Strategy

1	Möbius	1925
2	Moebius	695
3	1 OR 2	2420
4	Poland	260687
5	Poland's	823
6	4 OR 5	260687
7	3 AND 6	103
8	Poland-Möbius	17
9	Poland Möbius	79
10	Möbius-Poland	2
11	Poland-Moebius	10
12	Moebius Poland	36
13	Poland Moebius	36
14	Moebius-Poland	5
15	7 OR 8 OR 9 OR 10 OR 11 OR 12 OR 13 OR 14	103
16	15 AND Filters: MEDLINE	71

Accessed 16th November 2021
